# P17 - A frequently encountered condition: a case of urticaria multiforme

**DOI:** 10.1186/2045-7022-4-S1-P72

**Published:** 2014-02-28

**Authors:** Nazlı Cörüt, İlknur Bostancı

**Affiliations:** 1 Department of Pediatric Immunology and Allergy, Dr. Sami Ulus Gynecology and Pediatrics Training and Research Hospital, Ankara, Turkey

## Introduction

Urticaria multiforme observed in pediatric patients is a morphological sub-type of acute urticaria. It consists of acute, transient, annular and polycyclic erythematous papules and plaques that do not fade when pressed. We present an eight-month-old case with urticaria multiforme.

## Case report

An eight-month-old girl with fever, coughing, loss of appetite and pharyngeal hyperemia on going for five days was started on amoxicillin+clavulanic acid, but developed erythematous pruritic eruptions as well as swellings on the dorsum of her hands and feet on the fifth day of drug use. The family applied to the emergency service when the eruptions increased and began to turn purple. The infant’s temperature was determined as 38-38.5º C, and polycyclic papules and plaques that did not fade when pressed were observed. Some of these lession had echymotic centers and edema was identified on the dorsum of the hands and feet. Positive laboratory findings, CMV IgM and CMV IgG to be positive and C-reactive protein and a white blood cell count were high. The patient was referred to our department of pediatric immunology and allergy by doctors at the emergency service with a prediagnosis for erythema multiforme and serum-like sickness. Based on patient history and the physical examination findings, the patient was considered as having urticaria multiforme secondary to a cytomegalovirus infection. Previous medication were discontinued and was started on first generation antihistamines. She would fully recover within a period of approximately one week. The lesions had completely vanished on the tenth day of follow-up.

## Discussion

Urticaria multiforme is frequently misdiagnosed as a serum-like sickness or as urticarial vasculitis and erythema multiforme. These three diagnoses in question represent clinically different entities, each with their own specific treatment and prognosis. To ensure best patient care, pediatricians should be able to distinguish conditions that clinically mimic urticaria multiforme.

